# Multi-electrolyte-step anodic aluminum oxide method for the fabrication of self-organized nanochannel arrays

**DOI:** 10.1186/1556-276X-7-122

**Published:** 2012-02-14

**Authors:** Chun-Ko Chen, Sheng-Hui Chen

**Affiliations:** 1 Department of Optics and Photonics, National Central University, 300 Chung-Da Rd., Chung-Li, Taoyuan, 320, Taiwan

**Keywords:** nanochannel array, multi-electrolyte-step, anodic aluminum oxide

## Abstract

Nanochannel arrays were fabricated by the self-organized multi-electrolyte-step anodic aluminum oxide [AAO] method in this study. The anodization conditions used in the multi-electrolyte-step AAO method included a phosphoric acid solution as the electrolyte and an applied high voltage. There was a change in the phosphoric acid by the oxalic acid solution as the electrolyte and the applied low voltage. This method was used to produce self-organized nanochannel arrays with good regularity and circularity, meaning less power loss and processing time than with the multi-step AAO method.

## Introduction

In recent years, nanochannel arrays with periodic structures have been fabricated by various processes for application in several types of optical devices, such as in optical sensors [1], 2D photonic crystals [2], carbon nanotube field emission displays [3], nanowires [4-6], LED [7], and nanophotonics [8]. The anodic aluminum oxide [AAO] fabrication technique is one of the key methods for the fabrication of nanochannel arrays [9-11]. AAO nanochannel arrays with an interpore distance ranging from 50 to 420 nm have been obtained by anodizing aluminum in sulfuric, oxalic, and phosphoric acid solutions [12]. The advantages of the AAO process are the large area, high aspect ratio, simple process, and low cost. The self-organized multi-step AAO method has been applied for the fabrication of AAO nanochannel arrays with high uniformity [13-15]. The initial thickness of aluminum used in the multi-step AAO method is greater than 6 μm, especially for high bias voltage, large interpore distance nanochannel arrays. However, if the initial aluminum is the thin-film type, it is very difficult to deposit an aluminum film thicker than 10 μm without defects in the structure. In this paper, a novel process, the self-organized multi-electrolyte-step AAO method, is proposed for the growth of nanochannels with high and low applied voltages in the phosphoric and oxalic acid, respectively. This method can achieve nanochannel arrays with good regularity and circularity with less power loss and processing time than with the multi-step AAO method.

### Experiments

Before the AAO process, we have to make sure that the surface roughness of the aluminum foil is small enough for the growth of the nanochannel arrays [16]. High purity (99.99%) aluminum sheets were degreased in 5% NaOH for 30 s at 60°C and cleaned in a 1:1 volume mixture of nitric acid and deionized water. The aluminum was subsequently annealed at 400°C for 3 h and then electropolished in a mixture of H_2_SO_4_:H_3_PO_4_:H_2_O (ratio 2:2:1) at room temperature under a constant input current. After about 30 min of polishing, the mean roughness of the polished surface was measured by atomic force microscopy. The surface roughness was found to be reduced to approximately 3 nm in a 3-μm^2 ^scan area. The aluminum sheet was then mounted on a stainless steel that served as the anode. A graphite bar was used as the counter electrode. The anodization conditions involved different acid solutions as the electrolyte and different applied voltages under a temperature of 3°C. During the anodization process, an Al_2_O_3 _layer formed easily on the surface of the aluminum. Al^3+ ^and O^2- ^ions were dissociated due to the adding bias. Al^3+ ^reacts with the acid solution to make sure that Al^3+ ^will not combine with O^2-^, and an Al_2_O_3 _layer again formed on the surface of the aluminum. The repetition of the oxidization reaction and removal formed the self-organized nanochannels following the electrofield from the surface to the bottom of the substrate.

### Multi-step and multi-electrolyte-step AAO methods

**Figure 1:**
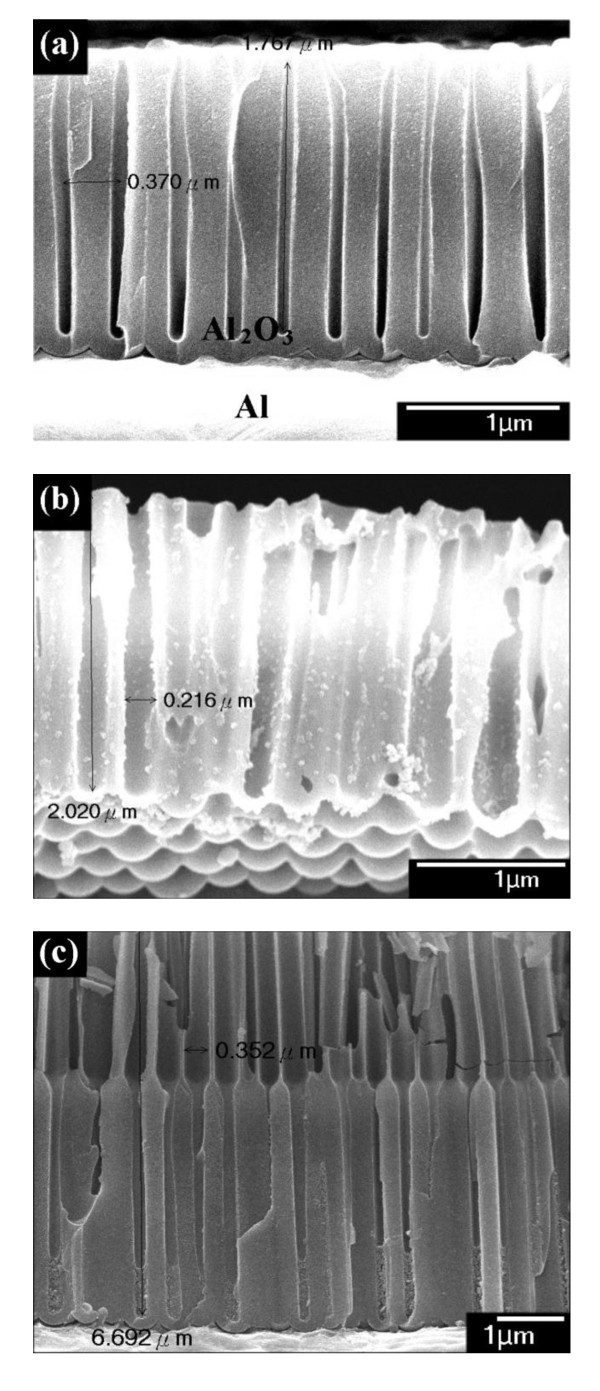
**Nanochannels formed by multi-step AAO**. **(a) **The first-step AAO nanochannels, **(b) **the expanded interpores, and **(c) **the second-step AAO nanochannels.

A five-step self-organized AAO method is first used to grow a nanochannel array. Figure 1a shows the scanning electron microscopic [SEM] image of the first-step AAO nanochannels. Then, the interpores of AAO were expanded by chemical dissolution in a 1-M NaOH solution for 8 min, as shown in Figure 1b. In the next step of the AAO process, the nanochannels will grow along the bottom of the first nanochannels, as shown in Figure 1c. The second AAO nanochannels are more regular than the first AAO nanochannels.

**Figure 2:**
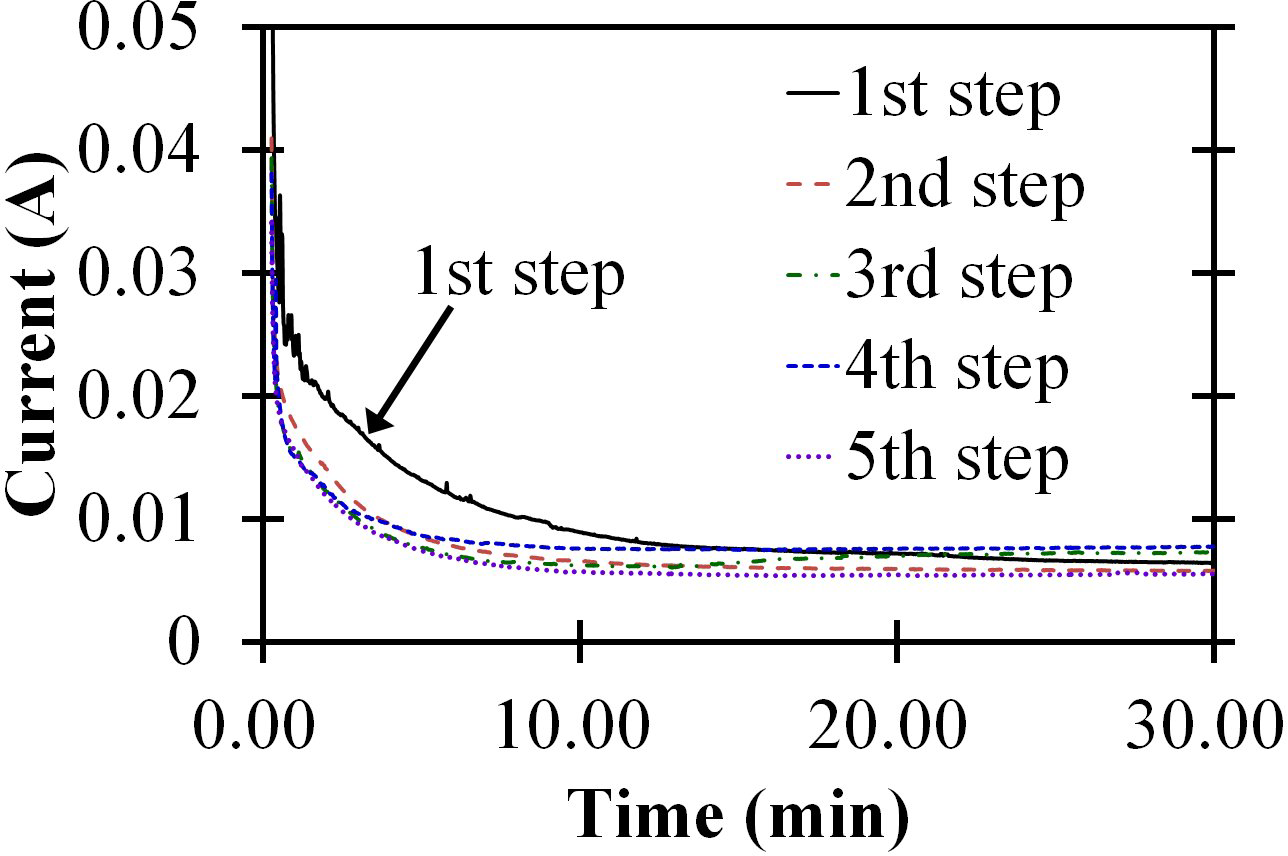
**Current-time curve of one and two- to five-step AAO process under 120V bias voltage**.

In order to ensure that the self-organized AAO process was stable enough in the experiments, a motorized system was applied to monitor the real-time current during the process. Figure 2 shows the current-time curve of the one- to five-step AAO process under the 120-V bias voltage. In the beginning of every step, the oxide layer would increase gradually along the surface of the AAO nanochannels leading to larger resistance. The current curves were reduced following the change in the resistance. After the first 5 min, the curves were in the stable range which was less than **±**1 mA. This means that the AAO nanochannel growth had reached a stable state with the electrochemical reaction. In addition, the current is larger in the first step than in the other steps, as shown in Figure 2; that is because the aluminum oxide is thin to lead to less resistance.

**Figure 3:**
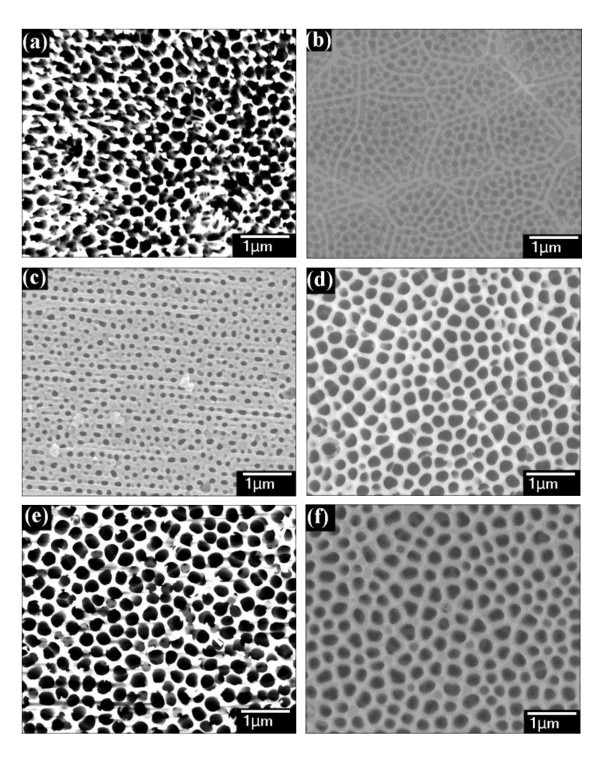
**SEM images of AAO nanochannels after different steps and applied voltages**. **(a) **100 V, one-step; **(b) **100 V, five-step; **(c) **120 V, one-step; **(d) **120 V, five-step; **(e) **140 V, one-step; and **(f) **140 V, five-step.

The anodization conditions of the multi-step AAO method were as follows: a 10-wt.% phosphoric acid solution was used as the electrolyte, and the applied voltages were 100 V, 120 V, and 140 V for 30 min. After anodization, the porous oxide films were removed by being submerged in a 0.5-M NaOH solution for 8 min. Figure 3 shows SEM images of the multi-step AAO nanochannels with different steps and applied voltages: (a) 100 V, one-step; (b) 100 V, five-step; (c) 120 V, one-step; (d) 120 V, five-step; (e) 140 V, one-step; and (f) 140 V, five-step. We can find that, after the five-step process, the self-organized nanochannel array behaved with better regularity under all of the applied voltage conditions. In addition, as the applied voltage became higher, the average period and the interpore diameter of the self-organized nanochannel array got larger.

**Figure 4:**
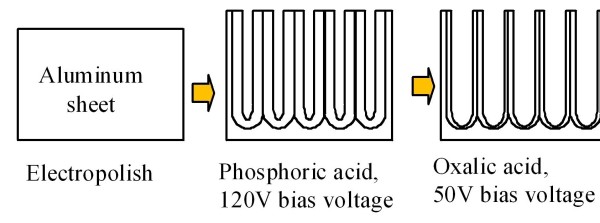
**Multi-electrolyte-step AAO process**.

The anodization conditions of the multi-electrolyte-step AAO method were as follows: a 1-wt.% phosphoric acid solution was used as the electrolyte, and the applied voltage was 120 V for a few minutes. Then, the phosphoric acid solution was replaced by an oxalic acid solution as the electrolyte, and an applied voltage of 50 V for 30 min was used. Figure 4 shows a flowchart of the multi-electrolyte-step AAO method.

### AAO quality analysis

**Figure 5:**
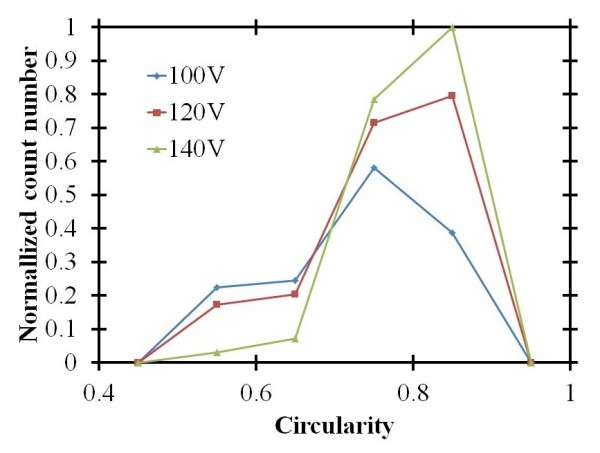
**Normalized count number of the self-organized nanochannel arrays with different circularities for different applied voltages**. Obtained using the multi-step AAO method.

The most important parameters for a self-organized nanochannel array are the diameter distribution of the interpores and the average period. However, the quality of the nanochannel array is hard to define. If the nanochannel array is arranged with better regularity, the nanochannels will be more similar to a circle. To analyze the quality of the self-organized nanochannel array, the circularity parameter can be applied. The definition of circularity *C *is as follows:

(1) $$C=4\pi \frac{A}{L^{2}},$$

where *A *and *L *are the area and the perimeter of the nanochannel, respectively. When the circularity value is 1.0, it indicates a perfect circle. As the value approaches zero, it indicates an increasingly elongated polygon. Figure 3a, b, c, d, e shows SEM images of the self-organized nanochannel arrays analyzed using the image processing software Image J (NIH, Bethesda, MD, USA) [17]. Figure 5 shows the normalized count number of the self-organized nanochannel arrays with different circularities for different applied voltages obtained using the one-step AAO method. The average circularities are 0.729, 0.763, and 0.796 when the applied voltages are 100 V, 120 V, and 140 V, respectively. We can see that the circularity increases as the applied voltage increases. In other words, a higher applied voltage can improve the shape of the nanochannel array. However, the regularity cannot be improved by just using a high applied voltage.

**Figure 6:**
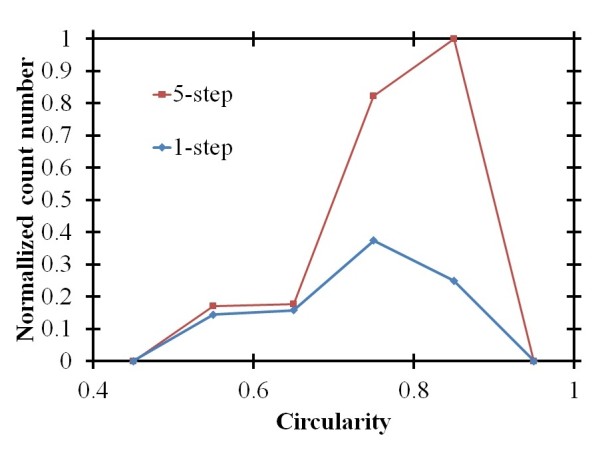
**Normalized count number of the self-organized nanochannel arrays with different circularities for the different steps**. Obtained using the multi-step AAO method.

Then we analyzed the circularity of the nanochannel arrays obtained with different AAO steps. Figure 6 shows the normalized count number of the self-organized nanochannel arrays with different circularities and an applied voltage of 100 V for the one- and five-step processes. From the distribution of the circularity, we find that the AAO nanochannels showed very good improvement of circularity in the five-step rather than the one-step AAO process. Comparison of the results in Figures 5 and 6 makes it very clear that the multi-step AAO method can improve the regularity more easily than the high applied voltage.

**Figure 7:**
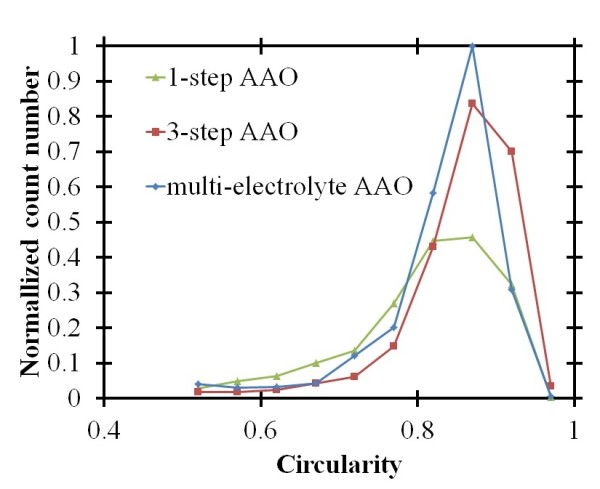
**Normalized count number of the self-organized nanochannel arrays with different circularities for the different methods**. Obtained with the one-step, three-step and multi-electrolyte-step AAO methods.

Finally, the multi-electrolyte-step AAO method is also analyzed and compared with the multi-step AAO method. A flowchart of the fabrication process is shown in Figure 4. At the beginning of the process, the self-organized nanochannels grew with a large period, 202 nm. A phosphoric acid electrolyte was used with an applied voltage of 120 V for a few minutes. After anodization, the porous oxide films were removed by being submerged in a 0.5-M NaOH solution for 8 min to achieve the nanobowl structures. Then, the nanochannel arrays grew again from the center of the nanobowl structure in the oxalic acid solution with 50 V of applied voltage. Figure 7 shows the normalized count number of the self-organized nanochannel arrays with different circularities obtained with the one-step, three-step, and multi-electrolyte-step AAO methods. The three-step and multi-electrolyte-step AAO methods can achieve better circularity distribution at about 0.87, which is higher than the one-step AAO method of 0.85. If we consider all of the process parameters, we can find that the initial thickness of the aluminum for the multi-step AAO method is about 6 μm which is thicker than the thickness obtained with the multi-electrolyte-step AAO method, which is about 2 μm.

**Figure 8:**
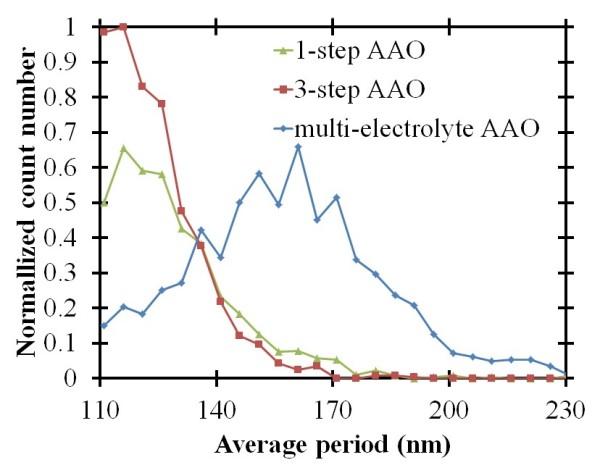
**Normalized count number of the self-organized nanochannel arrays for different average diameters**. Obtained with one-step, three-step, and multi-electrolyte-step AAO methods.

Table 1 shows all the parameters for the self-organized nanochannel arrays obtained with one-step, multi-step, and multi-electrolyte-step AAO methods. The periods and the diameters of the interpores can also be analyzed using image processing. The periods of the nanochannel arrays can be calculated from the FFT image of the SEM images [16]. The average periods are 203 ± 35 nm, 206 ± 15 nm, and 202 ± 20 nm for the one-step, multi-step, and multi-electrolyte-step AAO methods, respectively. Because the periods are all controlled by the first applied voltage, the results are similar. However, the one-step method is the most irregular of all the methods. Analysis of the diameters of the interpores, as seen in Figure 8, shows the normalized count number of the self-organized nanochannel arrays of the average diameters obtained with the one-step, three-step, and multi-electrolyte-step AAO methods. We can find that the average diameter is largest with the multi-electrolyte-step AAO method because a lower applied voltage can provide a thinner Al_2_O_3 _barrier layer. Table 1 shows a comparison of the results of the one-step, multi-step, and multi-electrolyte-step methods. It can be seen that the multi-electrolyte-step AAO method has the advantages of a shorter processing time, high quality of nanochannel, and low applied power.

**Table 1:** Parameters of the self-organized nanochannel arrays

	Method		
	One-step	Three-step	Multi-electrolyte
Step	one	three	approximately one
Al thickness	approximately 2 μm	approximately 6 μm	approximately 2 μm
Electrolyte	H_3_PO_4_	H_3_PO_4_	H_2_C_2_O_4_
Bias voltage	120 V	120 V	50 V
Processing time	30 min	90 min	approximately 30 min
Applied power	approximately 72 W	approximately 216 W	approximately 39 W
Circularity ± Δ	0.85 ± 0.1	0.87 ± 0.05	0.87 ± 0.05
Diameter	126.5 nm	122.4 nm	155.9 nm
Period ± Δ	203 ± 35 nm	206 ± 15 nm	202 ± 20 nm

The nanochannel arrays were obtained with the one-step, three-step, and multi-electrolyte-step AAO methods.

## Conclusion

In this study, nanochannel arrays are fabricated by the self-organized multi-step and multi-electrolyte-step AAO methods. The results show that, with the three-step and multi-electrolyte-step AAO methods, we can achieve better circularity distribution at about 0.87. However, the initial thickness of aluminum for the multi-electrolyte-step AAO method is about 2 μm, which is thinner than the thickness for the multi-step AAO method, 6 μm. Besides, the multi-electrolyte-step AAO method has the advantages of shorter processing time, high quality of nanochannels, and low applied power. Finally, self-organized nanochannel arrays fabricated by the multi-electrolyte-step AAO method show good circularity, large average diameters, and similar periods to those fabricated with the multi-step AAO method.

## Competing interests

The authors declare that they have no competing interests.

## Authors' contributions

S-HC conceived of the experiments, carried out the analyses, and drafted the manuscript. C-KC set up and performed the experiments. All authors read and approved the final manuscript.
